# Patient Preferences for Strategies to Improve Tuberculosis Diagnostic Services in Zambia

**DOI:** 10.1001/jamanetworkopen.2022.29091

**Published:** 2022-08-29

**Authors:** Andrew D. Kerkhoff, Lophina Chilukutu, Sarah Nyangu, Mary Kagujje, Kondwelani Mateyo, Nsala Sanjase, Ingrid Eshun-Wilson, Elvin H. Geng, Diane V. Havlir, Monde Muyoyeta

**Affiliations:** 1 Division of HIV, Infectious Diseases, and Global Medicine, Department of Medicine, Zuckerberg San Francisco General Hospital and Trauma Center, University of California, San Francisco School of Medicine, San Francisco; 2 Centre for Infectious Disease Research in Zambia, Lusaka, Zambia; 3 Department of Internal Medicine, University Teaching Hospital, Lusaka, Zambia; 4 Division of Infectious Diseases, Washington University School of Medicine, St Louis, Missouri

## Abstract

**Importance:**

Delayed engagement in tuberculosis (TB) services is associated with ongoing transmission and poor clinical outcomes.

**Objective:**

To assess whether patients with TB have differential preferences for strategies to improve the public health reach of TB diagnostic services.

**Design, Setting, and Participants:**

A cross-sectional study was undertaken in which a discrete choice experiment (DCE) was administered between September 18, 2019, and January 17, 2020, to 401 adults (>18 years of age) with microbiologically confirmed TB in Lusaka, Zambia. The DCE had 7 attributes with 2 to 3 levels per attribute related to TB service enhancements. Latent class analysis was used to identify segments of participants with unique preferences. Multiscenario simulations were used to estimate shares of preferences for different TB service improvement strategies.

**Main Outcomes and Measures:**

The main outcomes were patient preference archetypes and estimated shares of preferences for different strategies to improve TB diagnostic services. Collected data were analyzed between January 3, 2022, to July 2, 2022.

**Results:**

Among 326 adults with TB (median [IQR] age, 34 [27-42] years; 217 [66.8%] male; 158 [48.8%] HIV positive), 3 groups with distinct preferences for TB service improvements were identified. Group 1 (192 participants [58.9%]) preferred a facility that offered same-day TB test results, shorter wait times, and financial incentives for testing. Group 2 (83 participants [25.4%]) preferred a facility that provided same-day TB results, had greater privacy, and was closer to home. Group 3 (51 participants [15.6%]) had no strong preferences for service improvements and had negative preferences for receiving telephone-based TB test results. Groups 1 and 2 were more likely to report at least a 4-week delay in seeking health care for their current TB episode compared with group 3 (29 [51.3%] in group 1, 95 [35.8%] in group 2, and 10 [19.6%] in group 3; *P* < .001). Strategies to improve TB diagnostic services most preferred by all participants were same-day TB test results alone (shares of preference, 69.9%) and combined with a small financial testing incentive (shares of preference, 79.3%), shortened wait times (shares of preference, 76.1%), or greater privacy (shares of preference, 75.0%). However, the most preferred service improvement strategies differed substantially by group.

**Conclusions and Relevance:**

In this study, patients with TB had heterogenous preferences for TB diagnostic service improvements associated with differential health care–seeking behavior. Tailored strategies that incorporate features most valued by persons with undiagnosed TB, including same-day results, financial incentives, and greater privacy, may optimize reach by overcoming key barriers to timely TB care engagement.

## Key Points

**Question:**

Do patients with tuberculosis (TB) have differential preferences for strategies to improve TB diagnostic services?

**Findings:**

In this cross-sectional study of 326 patients newly diagnosed with TB in Lusaka, Zambia, 3 groups with distinct preferences for different TB diagnostic service enhancements were identified. Groups 1 and 2 were far more likely to report waiting at least 4 weeks after symptom onset to seek care, and they demonstrated strong preferences for facilities providing same-day TB test results, financial incentives, and greater privacy.

**Meaning:**

Tailored strategies that include features most preferred by persons with undiagnosed TB, who are likely to delay care seeking, have the potential to accelerate TB diagnosis and care engagement.

## Teaser

This cross-sectional study of patients with tuberculosis in Zambia examines preferences for improving tuberculosis diagnostic services.

## Introduction

Missed tuberculosis (TB) diagnoses represent the largest gap in the global TB care cascade.^1^ Because of the COVID-19 pandemic, this gap has been further widened, with an 18% decrease in notifications reported in 2020 compared with 2019.^1^ Furthermore, this gap does not reflect the substantial delays that many persons with TB may face to having their condition diagnosed and beginning treatment. Collectively, delayed and missed TB diagnoses contribute to morbidity, catastrophic economic costs, and mortality among socioeconomically vulnerable individuals and propagate continued transmission, undermining progress toward global control TB targets.^2,3,4^

Persons with TB living in high-burden, limited-resource settings may face several barriers at each step of their TB care journey, resulting in prolonged and complex care pathways.^5,6^ In most settings, patient-related delays in care seeking after symptom onset is the primary driver of delays in the overall TB care pathway; such delays are therefore an important area of focus for future TB interventions and program initiatives.^3,7,8^ To close existing gaps in TB detection and expedite care engagement, it is crucial that interventions and implementation strategies to reach persons with undiagnosed TB directly account for their differential barriers to engaging in TB services and their unique preferences by including them as stakeholders in the design process.

We previously reported the results of a discrete choice experiment (DCE) among patients with TB in Lusaka, Zambia, evaluating their preferences for implementing possible TB service improvement strategies that could accelerate TB diagnosis and care engagement by overcoming barriers to seeking and accessing health care.^8^ Overall, same-day TB test results, facilities with shorter wait times, and services closer to home were single-component strategies strongly preferred by most patients; no differences by HIV status or sex were found. However, exploring heterogeneity of preferences is often undertaken to optimize market shares (eg, reach) in commercial applications, and similar approaches could be applied to also enhance public health reach. It is unknown whether TB diagnosis and care engagement strategies may need to be tailored to appeal to distinct groups of patients with unique behaviors and preferences, including which strategies may be most preferred among persons with undiagnosed TB who are likely to substantially delay initial care engagement. Therefore, we undertook a latent class analysis of a DCE and simulated the predicted preferences of distinct TB patient preference groups for the hypothetical implementation of different single-component and multicomponent strategies to improve TB diagnostic services.

## Methods

### Setting and Participants

A prospective cross-sectional study of 401 patients with TB was undertaken between September 18, 2019, and January 17, 2020, at 2 public health facilities in Lusaka, Zambia.^8^ Individuals eligible for study inclusion were those 18 years or older, who had microbiologically confirmed TB, who had their TB episode classified as a new or relapse case, and who began anti-TB therapy within the prior 2 weeks.^8^ Ethical approval was obtained from the University of Zambia Biomedical Research Ethics Committee and the institutional review board of the University of California, San Francisco. All participants provided written informed consent in their preferred language. This study is reported in accordance with the Strengthening the Reporting of Observational Studies in Epidemiology (STROBE) reporting guideline.^9^

### DCE Procedures and Design

All eligible participants completed a structured survey followed by a DCE.^8^ The DCE was developed to determine patient preferences for improving characteristics of TB diagnostic services to inform the design of future strategies that could facilitate improved TB care engagement. The development and design process has previously been described in detail.^8^ The DCE attributes and levels were based on a review of the literature and refined through discussions with key stakeholders.^10^ Seven attributes with up to 3 attribute levels were included in the final design (Table 1). The final design was near balanced and orthogonal with respect to attributes and attribute levels (efficient design).^10,11^ All participants were shown a choice set with 9 random choice tasks.^8^ For each choice task, they were asked to choose which 1 of the 2 hypothetical TB diagnostic facilities that they most preferred based on an appraisal of differences in the health facility attribute levels; if neither option was acceptable, participants could select none.

**Table 1.** Discrete Choice Experiment Attributes and Levels for Evaluating Preferences of Patients With TB for Improving TB Diagnostic Services

Attribute	Level 1	Level 2	Level 3
Distance to the facility from participant’s home, km	2	6	10
Hours of facility operation	Open normal weekday hours	Open normal weekday hours and extended early morning or evening hours	Open normal weekday hours and Saturdays
Privacy and confidentiality of the facility	A place where no one knows the participant (enhanced privacy)	A place where the participant may be known or recognized (no enhanced privacy)	NA
Sex of health care workers at the facility	The health care worker is the same sex as participant	The health care worker may be either a man or a woman	NA
Total time spent at facility waiting and undergoing evaluation at the facility, h	2	5	8
TB test results (speed and notification method at the facility)	TB test results are available the same day before participant leaves	Participant will be contacted by telephone with TB test results and return instructions	Participant must return another day to the facility for TB test results
Financial incentive for undergoing TB testing and collecting the result at the facility, ZK	0	30 (approximately US $2)	60 (approximately US $4)

Abbreviations: NA, not applicable; TB, tuberculosis; ZK, Zambian kwacha.

### Statistical Analysis

All analyses were conducted between January 3, 2022, and July 2, 2022, using Lighthouse Studio, version 9.7.2 (Sawtooth Software) and Stata software, version 17.0 (StataCorp LLC). First, data cleaning was undertaken to improve the quality of individual-level data (eMethods 1 in the Supplement).^12,13,14^ Second, latent class multinomial logit was used to identify segments of participants with unique preferences for enhancing TB diagnostic services (eMethods 2 and eTable 1 in the Supplement).^12,13,15,16,17,18,19^ Third, a hierarchical bayesian model was used to calculate mean preference weights (ie, part-worth utilities) of attribute levels, overall and within each preference group (eMethods 3 in the Supplement).^12^ Sociodemographic details and health care–seeking delays after TB symptom onset were compared across preference groups using Fisher exact tests, Pearson χ^2^ tests, or Kruskal-Wallis tests as appropriate. Fourth, we sought to determine patient preferences for implementing different potential TB care engagement strategies and whether these differed by preference group. Individual participants’ mean preference weights were input into the Sawtooth Choice Simulator tool to estimate the shares of preference that different hypothetical enhanced health facilities would be expected to garner compared with a usual care facility among each of the 3 preference groups (eMethods 4 in the Supplement).^13^ Labels to succinctly describe key drivers of preference among each preference group were assigned based on simulation results. Sensitivity analyses were undertaken to evaluate the effect of different implementation contexts that differed according to the amount of total time spent at a facility and how far a facility was from a participant’s home and uncertainty around individual preference weight estimates. All statistical tests were 2-sided at α = .05.

## Results

Of 401 enrolled participants with newly diagnosed TB, 43 had incomplete DCE results and 32 had DCE data that fell below the root-likelihood fit statistic cutoff (eMethods 1 in the Supplement); therefore, 326 (81.3%) participants were included in the analysis. Overall, participants had a median age of 34 years (IQR, 27-42 years), 217 (66.8%) were male, 108 (33.2%) were female, and 158 (48.8%) were HIV positive (Table 2). A total of 194 (59.5%) were enrolled from a first-level hospital setting and 132 (40.5%) were enrolled from a tertiary hospital setting. Few differences were found between included and excluded participants (eTable 2 in the Supplement).

**Table 2.** Demographic and Socioeconomic Characteristics According to Latent Class Preference Group in the 326 Patients^a^

Characteristic	Overall (N = 326)	Group 1 (time is money) (n = 192)	Group 2 (privacy and convenience) (n = 83)	Group 3 (status quo) (n = 51)	*P* value^b^
Age, median (IQR), y	34 (27-42)	34 (27-43)	34 (27-42)	34 (25-41)	.63
Sex					
Male	217 (66.8)	116 (60.4)	60 (72.3)	41 (80.4)	.001
Female	108 (33.2)	76 (39.6)	22 (26.8)	10 (19.6)	
Educational level					
None or primary	141 (43.3)	84 (43.8)	33 (39.8)	24 (47.1)	.69
Secondary or tertiary	185 (56.8)	108 (56.3)	50 (60.2)	27 (52.9)	
Relationship status					
Currently married	155 (47.6)	83 (43.2)	44 (53.0)	28 (54.9)	.42
Divorced or separated	42 (12.9)	25 (13.0)	13 (15.7)	4 (7.8)	
Widowed	14 (4.3)	10 (5.2)	3 (3.6)	1 (2.0)	
Unmarried	115 (35.3)	74 (38.5)	23 (27.7)	18 (35.3)	
Religion					
Regularly go to church	135 (41.4)	87 (45.3)	35 (42.2)	13 (25.5)	.03
Sometimes go to church	120 (36.8)	68 (35.4)	33 (39.8)	19 (37.3)	
Not religious	71 (21.8)	37 (19.3)	15 (18.0)	19 (37.3)	
Primary income generator for household					
Yes	214 (65.9)	126 (65.6)	55 (66.3)	33 (66.0)	>.99
No	111 (34.2)	66 (34.4)	28 (33.7)	17 (34.0)	
Daily individual income, median (IQR), ZK	50 (20-100)	50 (0-100)	50 (30-100)	50 (27-100)	.15
HIV status					
Positive	158 (48.8)	107 (56.3)	31 (27.4)	20 (39.2)	.005
Negative	166 (51.2)	83 (43.7)	52 (62.7)	31 (60.8)	
History of smoking					
Yes, daily	120 (368)	56 (29.2)	33 (39.8)	31 (60.8)	.001
Yes, less than daily	28 (8.6)	18 (9.4)	9 (10.8)	1 (2.0)	
No	178 (54.6)	118 (61.5)	41 (49.4)	19 (37.3)	
Alcohol use disorder (positive AUDIT-C result)^c^					
Yes	191 (58.6)	99 (51.6)	52 (62.7)	40 (78.4)	.002
No	135 (41.4)	93 (48.4)	31 (37.4)	11 (21.6)	
Past TB treatment					
Yes	44 (13.5)	28 (14.6)	12 (14.5)	4 (7.8)	.47
No	282 (86.5)	164 (85.4)	71 (85.5)	47 (92.2)	
Knows someone who has been diagnosed with TB					
Yes, last 6 mo	55 (16.9)	28 (14.6)	13 (15.7)	14 (28.0)	<.001
Yes, but not last 6 mo	90 (27.7)	38 (19.8)	25 (30.1)	27 (54.0)	
No	180 (55.4)	126 (65.6)	45 (54.2)	9 (18.0)	

Abbreviations: AUDIT-C, Alcohol Use Disorders Identification Test–Concise; TB, tuberculosis; ZK, Zambian kwacha.

^a^ Data are presented as number (percentage) of patients unless otherwise indicated. Group 1 preferred a facility that offered same-day TB test results, shorter wait times, and financial incentives for testing. Group 2 preferred a facility that provided same-day TB test results, had greater privacy, and was closer to home. Group 3 had no strong preferences for service improvements and had negative preferences for receiving telephone-based TB test results.

^b^ *P* value tests whether there is a difference across the 3 groups using Fisher exact tests, Pearson χ^2^ tests, or Kruskal-Wallis tests, as appropriate.

^c^ Alcohol use disorder defined as an AUDIT-C score of 4 or higher in men and 3 or higher in women.

### Latent Class Groups According to Preferences for TB Service Improvement

Latent class analysis identified 3 segments that clustered according to distinct preferences for improving TB diagnostic services (Table 3). Group 1 (time is money) included 192 participants (58.9%); these individuals showed strong positive preferences for TB test results available the same day, shorter amounts of time spent at a facility, a facility closer to home, and small financial incentives for undergoing TB testing. Group 2 (convenience and confidentiality) included 83 participants (25.4%) who had strong positive preferences for a facility that provided same-day TB test results, a facility closer to home, and enhanced confidentiality and privacy. Group 3 (status quo) included 51 participants (15.6%) who did not have strong positive preferences for most TB diagnostic service enhancements but had strong negative preferences for a facility that provides TB testing results by telephone and a willingness to spend more time waiting and being evaluated.

**Table 3.** Mean Preference Weights for TB Service Attributes According to Latent Class Preference Group

Attribute	Mean preference weight (95% CI)			
	Overall (N = 326)	Group 1 (time is money) (n = 192)	Group 2 (privacy and convenience) (n = 83)	Group 3 (status quo) (n = 51)
Facility distance from home, km				
2	49.2 (44.4 to 54.0)	44.7 (38.4 to 51.1)	77.2 (73.2 to 81.2)	13.2 (11.1 to 15.3)
6	−1.9 (−4.0 to 0.1)	−8.8 (−11.8 to −5.8)	13.0 (8.6 to 17.4)	−10.8 (−14.3 to −7.3)
10	−47.3 (−52.8 to −41.8)	−36.0 (−42.5 to −29.4)	−90.2 (−96.7 to −83.7)	−2.4 (−6.5 to 1.7)
Confidentiality				
A place where no one knows who I am	16.7 (13.0 to 20.4)	15.6 (9.2 to 22.1)	37.1 (33.3 to 40.9)	−14.3 (−17.1 to −11.5)
A place where I may be known or recognized	−16.7 (−20.4 to −13.0)	−15.6 (−22.1 to −9.2)	−37.1 (−40.9 to −33.3)	14.3 (11.5 to 17.1)
Facility hours of operation				
Normal weekday hours	−3.7 (−5.5 to −1.9)	−4.0 (−7.3 to −0.7)	−9.2 (−13.4 to −5.0)	1.1 (−0.8 to 3.1)
Normal weekday hours and extra morning and evening hours	−5.3 (−7.3 to −3.4)	−5.3 (−8.2 to −2.4)	−5.3 (−8.0 to −2.7)	−3.0 (−6.2 to 0.2)
Normal weekday hours and open on Saturdays	9.0 (7.3 to 10.7)	9.3 (6.5 to 12.1)	14.5 (11.2 to 17.9)	1.9 (−1.2 to 5.0)
Sex concordance of health care workers				
The health care worker is the same sex as me	−2.4 (−4.8 to 0)	0.8 (−3.2 to 4.7)	−4.7 (−7.9 to −1.6)	−1.4 (−4.8 to 2.0)
The health care worker may be a man or a woman	2.4 (0 to 4.8)	−0.8 (−4.7 to 3.2)	4.7 (1.6 to 7.9)	1.4 (−2.0 to 4.8)
Total time spent at facility (waiting and evaluation), h				
2	30.6 (22.9-38.2)	72.8 (64.6 to 81.1)	14.6 (10.7 to 18.5)	−79.3 (−87.8 to −70.7)
5	7.5 (5.2-9.8)	4.1 (0.4 to 7.8)	2.9 (−0.6 to 6.3)	5.5 (0.3 to 10.8)
8	−38.1 (−45.0 to −31.2)	−76.9 (−84.3 to −69.5)	−17.5 (−20.2 to −14.7)	73.7 (68.7 to 78.8)
Incentive for TB testing, ZK				
0 (US $0)	−29.4 (−32.8 to −25.9)	−33.6 (−39.7 to −27.5)	−23.2 (−28.0 to −18.3)	−5.6 (−10.4 to −0.7)
30 (Approximately US $2)	3.2 (1.5-4.9)	0.4 (−3.1 to 3.9)	6.9 (4.5 to 9.3)	−6.7 (−10.4 to −3.0)
60 (Approximately US $4)	26.1 (23.6 to 28.7)	33.2 (28.6 to 37.7)	16.2 (12.2 to 20.2)	12.3 (8.7 to 15.9)
TB testing results				
TB testing results available before you leave (same-day results)	108.3 (100.7 to 115.8)	95.3 (84.6 to 106.0)	145.4 (139.7 to 151.1)	63.1 (55.0 to 71.2)
Contacted by telephone with TB testing results and return instructions	−103.5 (−113.6 to −93.3)	−28.4 (−34.1 to −22.6)	−154.0 (−161.1 to −146.8)	−232.0 (−236.3 to −227.7)
Must return to clinic another day to collect TB test results	−4.8 (−15.4 to 5.9)	−66.9 (−75.8 to −58.0)	8.5 (5.7 to 11.3)	168.9 (161.3 to 176.6)

Abbreviations: TB, tuberculosis; ZK, Zambian kwacha.

### Sociodemographic Characteristics According to Latent Class Preference Group

Compared with groups 2 and 3, participants in group 1 were more likely to be female (76 [39.6%] vs 22 [26.8%] in group 2 and 10 [19.6%] in group 3) and HIV positive (107 [56.3%] vs 31 [27.4%] in group 2 and 20 [39.2%] in group 3) (Table 2). Group 3 participants were more likely to have a history of smoking (31 [60.8%] vs 56 [29.2%] in group 1 and 33 [39.8%] in group 2), to have alcohol use disorder (40 [78.4%] vs 99 [51.6%] in group 1 and 52 [62.7%] in group 2), to personally know others who had been diagnosed with TB (41 [82.0%] vs 66 [34.4%] in group 1 and 38 [45.8%] in group 2), and to not be religious (19 [37.3%] vs 37 [19.3%] in group 1 and 15 [18.0%] in group 2). No differences were found by preference group according to age, educational level, relationship status, past TB treatment, or income (Table 2).

### TB Health Care–Seeking Behaviors According to Latent Class Preference Group

**Figure 1.**
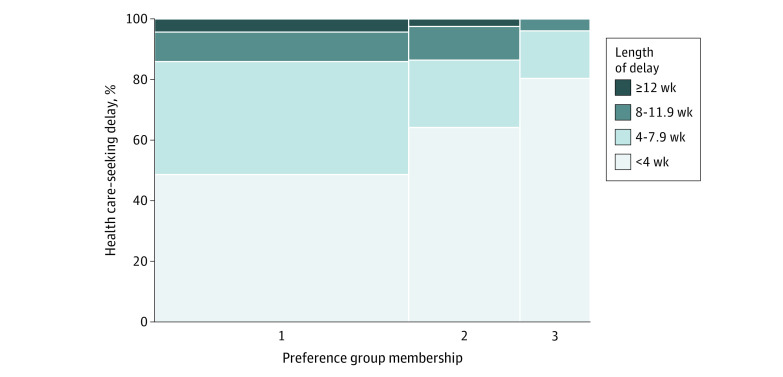
Health Care–Seeking Delays According to Latent Class Preference Group Membership (N = 326) Health care–seeking delays are defined as the time from first onset of any tuberculosis symptom to initial presentation to any practitioner and/or facility for evaluation of symptoms. The width of each column represents the relative proportion that each preference group accounts for of the overall study population.

Large differences were found in health care–seeking behaviors according to preference groups (Figure 1). Groups 1 and 2 were more likely to report at least a 4-week delay in seeking health care for their current TB episode compared with group 3 (95 [51.3%] in group 1, 29 [35.8%] in group 2, and 10 [19.6%] in group 3; *P* < .001). Furthermore, 26 (14.0%) of those in group 1 and 11 (13.6%) of those in group 2 had prolonged health care–seeking delays of 8 weeks or more compared with 2 (3.9%) of those in group 3, although this finding did not reach statistical significance (*P* = .13).

### Health Influences and Information Sources According to Latent Class Preference Group

Participants in group 1 were more likely to report that no one influenced their health decisions (81 [42.2%]) compared with groups 2 (21 [25.6%]) and 3 (4 [7.8%]). Among those reporting that certain persons influenced their health decisions, family members were the most influential (205 [93.6%]) and did not differ across groups (eTable 2 in the Supplement). However, groups 1 and 2 were far more likely than group 3 to report that friends (84 [75.7%] in group 1, 47 [77.1%] in group 2, and 27 [57.5%] in group 3), religious leaders (66 [59.5%] in group 1, 35 [57.4%] in group 2, and 17 [36.2%] in group 3), and health care workers (88 [79.3%] in group 1, 39 [63.9%] in group 2, and 11 [23.4%] in group 3) influenced their decisions (eTable 3 in the Supplement). Several channels were listed by participants as acceptable ways to reach them with health information, Trusted individuals, such as health care workers (308 [94.8%]), religious leaders (295 [90.8%], and teachers (309 [95.1%]), were more likely to be cited by participants than traditional and digital media forms, such as newspapers and magazines (195 [59.8%]), radio (281 [86.2%]), television (273 [83.7%]), billboards (259 [79.5%]), and social media (229 [70.3%]). Group 3 participants were more receptive to several different channels for being reached with health messages (eTable 3 in the Supplement).

### Preference Simulations for TB Diagnostic Service Improvements According to Latent Class

**Figure 2.**
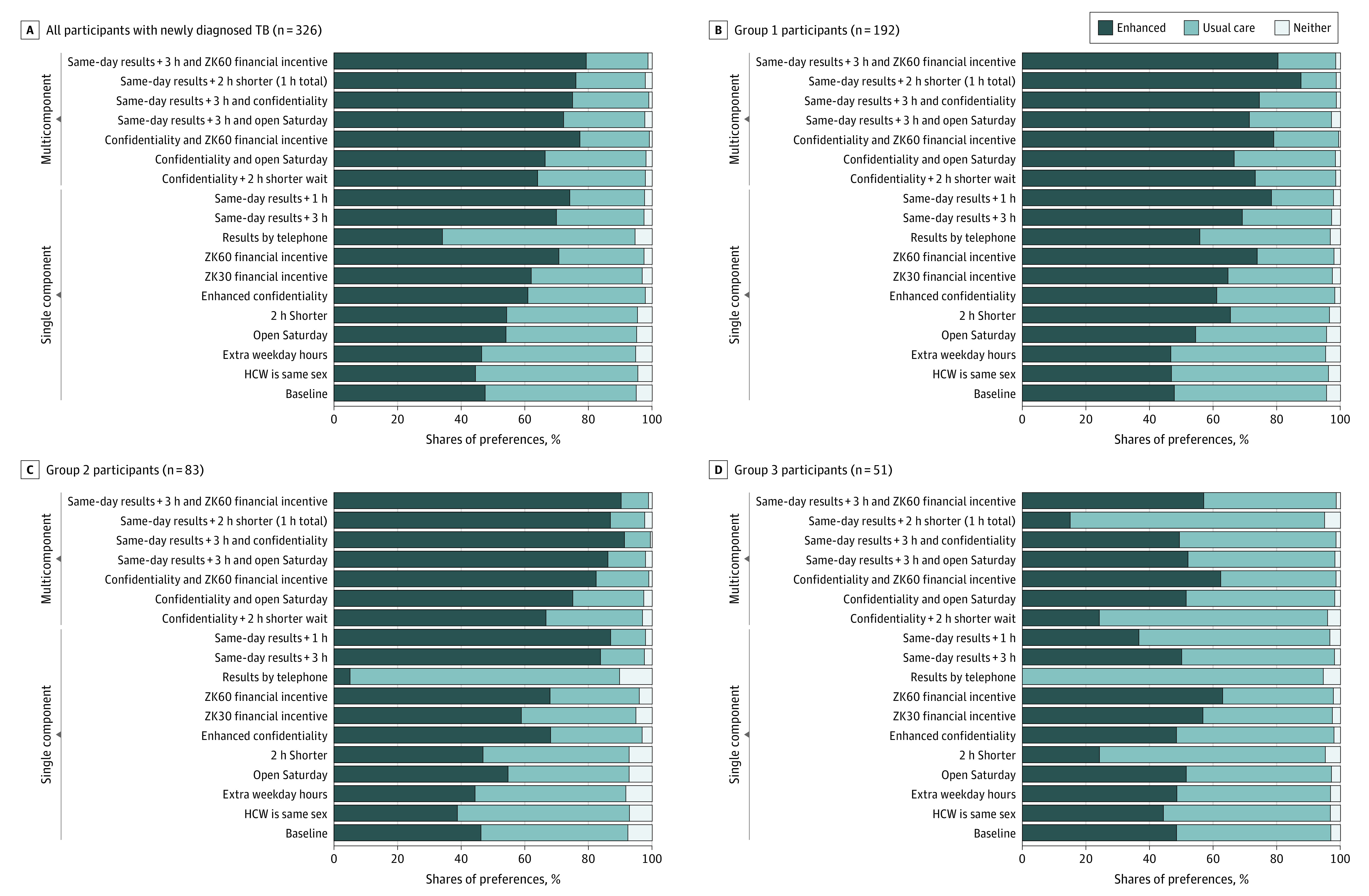
Preference for Single-Component and Multicomponent Strategies to Enhance Existing Tuberculosis (TB) Diagnostic Services Among All Participants (N = 326) Shares of preferences were estimated under varying assumptions for an enhanced facility, a usual care facility, or neither facility. The usual-care health facility parameters were based on the service features of an average TB diagnostic facility based at a first-level health facility in Lusaka, Zambia, which was assumed to be 2 km from a participant’s home, require 3 hours spent at the clinic waiting and undergoing evaluation (based on the median amount of time cited by survey participants on their date of TB diagnosis), only be open during typical business hours Monday through Friday, be a facility where an individual may be known or recognized, not offer sex-concordant health care workers (HCWs), not offer financial incentives for undergoing TB testing, and require patients to return on a different day to collect their TB test results. For each scenario, an enhanced facility was assumed to have the same features as the usual care facility with the exception that it offered 1 or more improved service features that were evaluated during the discrete choice experiment. ZK indicates Zambian kwacha (to convert to equivalent US dollars at the outset of study enrollment on September 19, 2019, multiply by 0.076).

We modeled the estimated shares of preference overall and by each latent class preference group for implementing single-component and multicomponent TB diagnostic service improvement strategies when compared with a usual-care TB diagnostic facility in Lusaka, Zambia (Figure 2). Overall, the most preferred strategies were same-day TB test results with a 1- to –3-hour additional wait time (shares of preference, 69.9%-74.1%), financial incentives (shares of preference, 62.0%-70.7%), and a facility offering enhanced privacy (share of preference, 61.0%) compared with a usual care facility (share of preference, 47.%) (Figure 2A); preferences were maximized among all participants by combining same-day test results with a ZK60 (Zambian kwacha; approximately US $4 at time of study enrollment) financial incentive for undergoing TB testing (share of preference, 79.3%). Among group 1 participants (time is money) (Figure 2B), a facility providing same-day TB test results with a 1-hour (share of preference, 69.2%) to 3-hour (share of preference, 72.4%) additional wait time, a financial incentive of ZK30 (share of preference, 64.7%) to ZK60 (share of preference, 74.0%), 2 hours shorter total visit time (share of preference, 65.5%), and enhanced confidentiality (share of preference, 61.2%) substantially increased preferences compared with the usual care facility (share of preference, 47.8%); combining these features further increased preferences for an enhanced facility, with the most preferred strategy being same-day test results combined with a ZK60 financial incentive (41 [80.4%]). Group 2 participants most preferred a facility offering same-day TB test results (shares of preference, 83.8%-87.0%) and had strong preferences for a facility offering enhanced privacy (share of preference, 68.1%) and a ZK30 to ZK60 financial incentive (shares of preference, 58.9%-67.9%) compared with a usual care facility (share of preference, 46.2%) (Figure 2C); they showed negative preferences for a facility providing TB test results by telephone (share of preference, 5.0%). The most preferred strategy among group 2 participants was a facility that provided same-day TB test results and enhanced confidentiality (share of preference, 91.4%). Among group 3 participants, only financial incentives were estimated to meaningfully increase preference shares compared with the usual care facility (shares of preference, 56.8%-63.0% vs 48.5%), whereas several features decreased preference shares (Figure 2D); notably, a facility providing TB test results by telephone was not estimated to be preferred by any group 3 participants.

Sensitivity analyses revealed that in a setting where a usual-care TB diagnostic facility was located farther from home (4 km vs 2 km), a strategy of providing diagnostic services closer to where participants lived substantially improved shares of preferences for all 3 preference groups (eTable 4 in the Supplement). Furthermore, in a setting where enhanced features could only be implemented at a centralized TB diagnostic facility, facilities providing Saturday hours, financial incentives for TB testing, and same-day TB test results increased shares of preferences despite a need for participants to travel 2 km farther to access such facilities (eTable 4 in the Supplement). In both sensitivity analyses, optimal strategies differed for groups 1, 2, and 3. A comparison of 2 different simulation models demonstrated similar shares of preference estimates for different service enhancement strategies (eTable 5 in the Supplement).

## Discussion

In this DCE from Lusaka, Zambia, we found that patients with TB had heterogenous preferences for strategies to improve TB diagnostic services that clustered into 3 distinct groups. The 3 groups not only differed according to sociodemographic characteristics but also had differential engagement behaviors, such that patients with TB in groups 1 and 2 were far more likely to delay seeking evaluation for their TB symptoms by more than 4 weeks. Large differences were also found between groups regarding trusted persons who influence health-related decisions and preferred sources for receiving health information. Although the implementation of same-day TB test results and small financial incentives were predicted to be highly preferred by most participants, single-component and multicomponent strategies that optimized preference differed substantially among the groups. Collectively, these results suggest that (1) there are unique archetypes of patients with TB that cannot be predicted by demographic characteristics alone; (2) not all patients with TB require the availability of enhanced features or strategies to seek care for their symptoms in a timely manner (eg, within 4 weeks); and (3) to overcome their unique barriers and reduce the time to initial health care seeking, implementation of tailored care engagement strategies may be required to reach and appeal to different archetypes of persons with undiagnosed TB who are more likely to have prolonged delays in seeking health care.^20^

Participants clustering into preference groups 1 (time is money) and 2 (convenience and confidentiality) were much more likely to delay seeking initial health care for their symptoms compared with those in group 3 (status quo). Strategies that could reach, engage, and provide TB diagnosis among group 1 and 2 archetypes sooner may not only improve individual clinical outcomes and reduce the economic costs associated with prolonged care pathways but also substantially drive down community transmission by reducing periods of infectiousness.^3,21^ The availability of convenient, same-day test results was highly preferred among participants in groups 1 and 2. However, the feasibility of delivering same-day TB test results at health care facilities in many high-burden settings is currently challenged by the limitations of available diagnostic tools (eg, Xpert) and their resource requirements.^22,23^ Investments should be made to develop accurate, point-of-care TB triage and diagnostic tools that can be scaled up in community and lower-level health care settings where persons with undiagnosed TB may first present.^24,25,26^ We also found that provision of convenient, decentralized TB diagnostic services would be highly valued to individuals who voiced strong positive preferences for services closer to their home (eg, groups 1 and 2) and services that reduce their time waiting and being evaluated.^27,28^ Such participants also showed strong positive preferences for small financial incentives to undertake TB testing. Monetary rewards conditional on undertaking a positive health behavior are feasible, acceptable, and effective at improving care engagement for HIV in resource-limited settings^29,30,31^; they could similarly increase motivation to seek care for TB symptoms sooner by positively shifting the opportunity costs associated with seeking health care. Furthermore, group 1 and 2 archetypes also indicated strong preferences for a diagnostic strategy that incorporated greater privacy, where no one would recognize them. This finding suggests that TB-related stigma remains a key barrier that should be addressed as part of multicomponent strategies to accelerate TB care engagement and improve TB diagnosis.^32^

In addition to differential preferences for TB diagnostic service improvements, group 1 and 2 archetypes had different trusted health influences and sources of information. Although family members were the most trusted source of health information across the 3 groups (>90%), friends were more likely to directly influence the health-related decision-making of groups 1 and 2. Thus, social network–based approaches in which patients with newly diagnosed TB directly reach out to their friends and family who are close contacts may represent a promising TB case–finding strategy.^33,34^ Religious leaders were also more likely to be cited as trusted persons who influenced the health-related decisions of groups 1 and 2. This finding suggests that trusted community members outside the health care system are important stakeholders to engage in the design and implementation of future TB control efforts to optimize reach.^35,36^

Group 3 comprised the smallest patient archetype, but most (>80%) of these participants reported seeking care within 4 weeks of TB symptom onset. This finding was somewhat surprising given that such persons were likely to have classically described TB risk factors, such as a history of smoking and alcohol use disorder, and may be expected to have poor health care–seeking behavior. However, participants in group 3 were much more likely to state that they personally knew at least 1 person who had previously been diagnosed with TB. Group 3 participants may therefore have had greater TB-related knowledge and TB risk perception, prompting earlier health care seeking after symptom onset. Although group 3 participants had weaker preferences for TB diagnostic service enhancements because they were the group most likely to seek care in a timely manner, it may be less important to account for their unique preferences, because usual care services appeared to largely meet their needs. Notably, we found that scaling up the delivery of TB test results by telephone would be completely unacceptable to this patient archetype and could unintentionally discourage already largely positive health care–seeking behaviors. It was not clear whether privacy concerns, lack of reliable access to a mobile telephone and/or data, or other factors drove strong negative preferences for this service feature among group 3 as well as group 2 participants. These findings require further exploration through qualitative research and provide a cautionary tale for intervention and program designers.

### Limitations

This study has some limitations. Patients with TB were enrolled from large facilities in Lusaka, a large urban population center that accounts for a large proportion of Zambia’s TB notifications^37^; however, health-related behaviors may be different among residents in rural settings, where geographic access and costs of seeking health care may serve as a stronger barrier to engaging in TB services. Furthermore, because surveys were administered in person with the help of a trained research assistant, some responses may have been subject to social desirability bias; however, DCEs are less prone to such bias given the indirect manner in which preferences are elicited.^13^ In addition, DCEs capture participants’ stated preferences under hypothetical conditions^38^; therefore, it cannot be known whether implementation of the most preferred strategies would translate into improved health care–seeking behaviors without additional studies conducted in community settings.^39,40^ Nonetheless, DCEs have been shown to have strong value for predicting actualized choices and represent an important tool for ensuring that health interventions and implementation strategies are aligned with the preferences of patients, community members, and other key stakeholders.^38,41^

## Conclusions

This study revealed multiple archetypes of patients with TB in Lusaka, Zambia, defined by distinct preferences for implementing different strategies to improve TB diagnostic services. These archetypes also identified groups of patients who were substantially more likely to delay seeking health care as well as differential trusted sources of health information. The delivery of tailored multicomponent interventions and implementation strategies accounting for known health care preferences and preferred communication channels may optimize reach and acceptability and ultimately facilitate improved and expedited TB diagnosis and care engagement in settings with high TB burdens.

## Notes

Supplement. **eMethods 1.** Description of Data Cleaning Procedures Undertaken to Improve the Quality of Individual-Level Respondent Data **eMethods 2.** Description of Latent Class Analysis Procedures for Identifying Distinct Preference Groups **eMethods 3.** Description of Hierarchical Bayes Analysis Procedures for Estimating Preference Weights **eMethods 4.** Description of Simulation Procedures for Estimating Shares of Preference **eTable 1.** Indices of Statistical Fit for Latent Class Solutions (2 to 5) for Identifying Groups of Tuberculosis Patients With Distinct Preferences Based on Discrete Choice Experiment Results **eTable 2.** Evaluation of Sociodemographic Characteristics and Health-Seeking Behaviors According to Whether the Participants Were Included in the Final Data Analysis (N=401) **eTable 3.** Health Influences According to Latent Class Preference Group (N=326) **eTable 4.** Simulated Shares of Preference (%) for a Health Facility Offering at Least One Enhanced Service Feature Under Three Different Implementation Scenarios According to Latent Class Preference Group* **eTable 5.** Simulated Shares of Preference Using Two Different Models for a Health Facility Offering at Least One Enhanced Service Feature Using Two Different Estimation Methods According to Latent Class Preference Group
eReferences
