# Supplementary material for: Patient Preferences for Strategies to Improve Tuberculosis Diagnostic Services in Zambia
Source: JAMA Netw Open. 2022 Aug 29;5(8):e2229091. doi: 10.1001/jamanetworkopen.2022.29091 (PMC9425150; doi:10.1001/jamanetworkopen.2022.29091)
Supplement: Supplement. — eMethods 1. Description of Data Cleaning Procedures Undertaken to Improve the Quality of Individual-Level Respondent Data eMethods 2. Description of Latent Class Analysis Procedures for Identifying Distinct Preference Groups eMethods 3. Description of Hierarchical Bayes Analysis Procedures for Estimating Preference Weights eMethods 4. Description of Simulation Procedures for Estimating Shares of Preference eTable 1. Indices of Statistical Fit for Latent Class Solutions (2 to 5) for Identifying Groups of Tuberculosis Patients With Distinct Preferences Based on Discrete Choice Experiment Results eTable 2. Evaluation of Sociodemographic Characteristics and Health-Seeking Behaviors According to Whether the Participants Were Included in the Final Data Analysis (N=401) eTable 3. Health Influences According to Latent Class Preference Group (N=326) eTable 4. Simulated Shares of Preference (%) for a Health Facility Offering at Least One Enhanced Service Feature Under Three Different Implementation Scenarios According to Latent Class Preference Group* eTable 5. Simulated Shares of Preference Using Two Different Models for a Health Facility Offering at Least One Enhanced Service Feature Using Two Different Estimation Methods According to Latent Class Preference Group eReferences [file jamanetwopen-e2229091-s001.pdf]

Supplementary Online Content

Kerkhoff AD, Chilukutu L, Nyangu S, et al. Patient preferences for strategies to improve tuberculosis diagnostic services in Zambia. *JAMA Netw Open*. 2022;5(8):e2229091.
doi:10.1001/jamanetworkopen.2022.29091

eMethods 1. Description of Data Cleaning Procedures Undertaken to Improve the Quality of Individual-Level Respondent Data
eMethods 2. Description of Latent Class Analysis Procedures for Identifying Distinct Preference Groups
eMethods 3. Description of Hierarchical Bayes Analysis Procedures for Estimating Preference Weights
eMethods 4. Description of Simulation Procedures for Estimating Shares of Preference
eTable 1. Indices of Statistical Fit for Latent Class Solutions (2 to 5) for Identifying Groups of Tuberculosis Patients With Distinct Preferences Based on Discrete Choice Experiment Results
eTable 2. Evaluation of Sociodemographic Characteristics and Health-Seeking Behaviors According to Whether the Participants Were Included in the Final Data Analysis (N=401)
eTable 3. Health Influences According to Latent Class Preference Group (N=326)
eTable 4. Simulated Shares of Preference (%) for a Health Facility Offering at Least One Enhanced Service Feature Under Three Different Implementation Scenarios According to Latent Class Preference Group*
eTable 5. Simulated Shares of Preference Using Two Different Models for a Health Facility Offering at Least One Enhanced Service Feature Using Two Different Estimation Methods According to Latent Class Preference Group
eReferences

This supplementary material has been provided by the authors to give readers additional information about their work.

© 2022 Kerkhoff AD et al. *JAMA Network Open.*

eMethods 1. Description of Data Cleaning Procedures Undertaken to Improve the Quality of Individual-Level Respondent Data

First, data was assessed to identify and remove any participants who chose the same option across choice tasks (e.g., straight-lining). Further, a simulation of 500 randomly-answering mock participants completing the final DCE design was undertaken to determine the upper 95% confidence limit cutoff of the root-likelihood fit statistic (RLH). Data from study participants falling below this cutoff (RLH=0.535) were excluded as it suggests that they may not have comprehended the DCE and/or responded randomly rather than thoughtfully valuating each choice task. Among 358 participants with complete DCE results, there were no 'straight-participants' identified, however, 32 participants had DCE data with RLH values below the RLH cutoff (<0.535) and they were therefore not included in the final analysis.

eMethods 2. Description of Latent Class Analysis Procedures for Identifying Distinct Preference Groups

Latent class analysis using a latent class multinomial logit model within Sawtooth Software was used to identify segments of participants with unique preferences for enhancing TB diagnostic services. Latent class analysis assumes that a sample is composed of groups (classes) that have identical preference weights and that differ systematically from preference weights in the other classes.[1] Participants' chosen product alternative for each random choice task define the dependent variable, while the attribute levels that make up each alternative in a random choice task define the independent variables. In latent class multinomial logit models, it is typically assumed that multiple responses (i.e., choices) from participants are independent and thus potential within-participant correlation is not controlled for.[1]

Latent class solutions ranging from two to five groups were computed through an iterative process to derive group preference weights (level variations in attributes) that best fit participants' choices for different product alternatives – as indicated by maximum likelihood.[2] First, for each group solution, random estimates of the groups' preference weight are assigned. Then these weights are used to estimate each participant's choice data and their probability of belonging to each class. These probabilities are then used to re-estimate the logit probability weights at which point the log-likelihood is summed across groups. These steps are then repeated until the log-likelihood no longer improves by >0.01 at which point convergence is achieved.[2] The latent class multinomial logit model was allowed to run up to 500 iterations (automatically starting from one of five different random starting points) before it reached the convergence limit for log-likelihood. From the final iteration, each participant's probability of belonging to each one of the three latent class groups was estimated (i.e., posterior probability), where the three values sum to 1. Each participant was then classified into one of the three preference groups based on their highest posterior probability. Group preference weights derived from the latent class analysis were not included in the final analysis, and overall, and group-specific preference weights were estimated using Hierarchical Bayes (see **Section C** below).

For the final model, the number of distinct preference groups was selected by considering, which solution optimized statistical fit, and the interpretability of preference archetypes represented by each group (**eTable 1**). Two, three, four and five group latent class solutions were evaluated. Since there is no consensus as to which single statistical criteria indicates the best model fit, we used several information criterion (IC) to evaluate each latent class solution.[3,4] This included Bayesian information criteria (BIC), which was the primary IC considered, as well as the Akaike information criterion (AIC) and Consistent Akaike information criterion (CAIC), where lower values for each measure indicate a better fit. Elbow plots of each fit statistic were constructed to visually evaluate at which class solution the fit changed. On this basis, three and four group solutions were then selected for further evaluation. Next, we evaluated the preference weights for each attribute level across the three and four group solutions to understand the different preference archetypes. The additional preference

© 2022 Kerkhoff AD et al. *JAMA Network Open.*

archetype group identified under a four-group solution was not clearly interpretable relative to three other preference groups. Further, a four-group solution resulted in a decrease in the average maximum membership probability relative to a three-group solution (0.874 versus 0.901), which indicated that the model did not perform as well in classifying each participant into a corresponding preference group. Therefore, a three-group latent class solution was selected as the final solution for further subsequent analyses.

© 2022 Kerkhoff AD et al. *JAMA Network Open.*

eMethods 3. Description of Hierarchical Bayes Analysis Procedures for Estimating Preference Weights

Prior to undertaking estimation of preference weights, for each random task, independent variables (i.e., attribute levels that define each alternative in a random choice task) were "effects-coded" to reflect the presence or absence of a feature in a product alternative and the dependent variable (i.e., chosen product alternative) was coded as a binary variable (1=chosen, 0=not chosen).[1,5] A hierarchical Bayesian (HB) model within Sawtooth Software was then used to calculate mean preference weights, overall, and within each preference group. Preference weights (also known as part-worth utilities) measure the relative utility (i.e., unique value) of a given attribute level (i.e., feature) for a participant and indicate how much that level influences a participant's decision making regarding the product (e.g., TB diagnostic service program). HB models have two levels: (1) a 'higher' level model that assumes each participant's preference are described by a multivariate normal distribution; and (2) a lower-level model that assumes that based on a participant's preference weights, their probability for choosing a product alternative are described by a multinomial logit model.[6] Overall, the HB algorithm seeks to balance the maximal individual likelihood ('lower level') times the probability from the multivariate normal distribution ('higher level'). The upper-level model estimates population mean parameters (**α =** a vector of means of the distribution of participants' preference weights) and covariance matrix (**D =** matrix of variances and covariances of the distribution of preference weights across all participants**)**. The lower-level model estimates preference weights (**ß** = a vector of preference weights for a given individual) that fit each participant's choice data as well as possible (e.g., their chosen concept for each random choice task [dependent variable] with varying attribute levels that define each product alternative [independent variables]).[6] The algorithm down-weights (shrinks) extreme preference weights from unusual or unstable respondents to provide stability and improve model predictions. Preference weights were derived through iterative estimation of parameters using Monte Carlo Markov Chain algorithm (Gibbs Sampler). Because DCE's rely on participants to answer multiple choice tasks, their responses (i.e., choices) may be more similar (e.g., within-participant correlation).[7] However, no formal correction for within-participant correlation was applied as there was extremely minimal evidence of clustering effect (individual-level intraclass correlation [ICC] = 0.03 [95%CI: 0.01-0.05]) and thus, would not be expected to meaningfully influence preference weight estimates. First, 10,000 iterations were run before convergence to a stable solution was assumed; convergence was also assessed through visual inspection. After convergence, 10,000 additional iterations (i.e., draws) were run and the actual draws of **ß** for each of the 10,000 iterations were averaged (mean) to estimate 'average preference weights' and their corresponding standard deviations and 95% confidence intervals. Preference weights were then scaled to sum to zero within each attribute (i.e., 'zero-centered preference weights'), such that positive and negative preference weights represent more positive and negative preferences for a given attribute level, respectively.

© 2022 Kerkhoff AD et al. *JAMA Network Open.*

eMethods 4. Description of Simulation Procedures for Estimating Shares of Preference

The Sawtooth Choice Simulator utilizing the shares of preference (logit rule) method was used to estimate the predicted shares of preference different hypothetical “enhanced” health facilities would be expected to garner compared to a “usual care” facility overall among all participants, and among each of the three preference groups. A shares of preference model was selected over other estimation approaches because it assumes that respondents do not always choose the product (i.e., the TB diagnostic service facility) that provides the highest utility and through a scaling procedure, may produce estimates that more closely mimic decision making in the real world.[2] This method uses the logit equation to estimate shares. First, individual participants’ average preference weights (estimated using Hierarchical Bayes as described in **Section C** above) were loaded into the Choice Simulator. To derive the “share of preference” for a hypothetical TB diagnostic facility, the total utility for each of the two facilities is first determined (e.g., the sum of preference weights for the levels that comprise the enhanced and usual care facility, respectively). Then the total utility for each of the two facilities undergoes exponential transformation (i.e., the antilog). Finally, the resulting values for each of the two facilities are rescaled so that they sum to 100. Of note, uncertainties for preference weight estimates were not directly accounted for within the shares of preference (logit rule) simulation method. However, mean preference weights were derived by taking the average of 10,000 ‘draws’ (as described in **section C** above), which substantially reduces uncertainty around estimates. To evaluate how the inclusion of uncertainty around preference weights may affect shares of preference estimates derived from simulations, we undertook a sensitivity analysis using a Randomized First Choice (RFC) Model. The RFC model first adds random error to preference weights and then assumes that participants will choose the facility associated with the highest utility.[2] For each evaluation between two facilities (e.g., enhanced and usual care), 250,000 total iterations (~767 per participant) were run in which participants were simulated to make repeated first choices between the two facilities that incorporated random preference weight errors in each iteration to estimate the overall shares of preference.

© 2022 Kerkhoff AD et al. *JAMA Network Open.*

eTable 1. Indices of Statistical Fit for Latent Class Solutions (2 to 5) for Identifying Groups of Tuberculosis Patients With Distinct Preferences Based on Discrete Choice Experiment Results

	**BIC**	**AIC**	**CAIC**	**Average maximum membership probability***
**2 groups**	4443	4281	4470	0.962
**3 groups**	4403	4158	4444	0.901
**4 groups**	4398	4069	4453	0.874
5 groups	4433	4021	4502	0.881

Abbreviations: AIC= Akaike information criterion; BIC = Bayesian information criteria; CAIC = Consistent Akaike information criterion. *Average maximum membership probability corresponds to the average of each individual's highest posterior membership probability (of three) for a given preference group, which directly corresponds to the latent class group to which they were assigned. Please see Supplementary Appendix B for a detailed description of how the final latent class model was selected.

© 2022 Kerkhoff AD et al. *JAMA Network Open.*

eTable 2. Evaluation of Sociodemographic Characteristics and Health-Seeking Behaviors According to Whether the Participants Were Included in the Final Data Analysis (N=401)

	**Included in final analysis (n=326)** **N (%)**	**Not included in final analysis (n=75)** **N (%)**	**P-value**
**Age, median (interquartile range)**	34 (27-42)	33 (25-43)	0.58
**Gender**			
Male	217 (66.8)	58 (77.3)	0.08
Female	108 (33.2)	17 (22.7)	
**Education**			
None/primary	141 (43.3)	31 (41.3)	0.76
Secondary/tertiary	185 (56.8)	44 (58.7)	
**Relationship status**			
Currently married	155 (47.6)	37 (49.3)	0.84
Divorced or separated	42 (12.9)	7 (9.3)	
Widowed	14 (4.3)	4 (5.3)	
Unmarried	115 (35.3)	27 (36.0)	
**Faith**			
Regularly go to church	135 (41.4)	34 (45.3)	0.81
Sometimes go to church	120 (36.8)	25 (33.3)	
Not religious	71 (21.8)	16 (21.3)	
**Primary income generator for household**			
Yes	214 (65.9)	53 (70.7)	0.42
No	111 (34.2)	22 (29.3)	
**Daily individual income (in Kwacha), median (interquartile range)**	50 (20-100)	30 (0-66)	0.019
**HIV-status**			
Positive	158 (48.8)	31 (41.3)	0.27
Negative	166 (51.2)	44 (58.7)	
**History of smoking**			
Yes, daily	120 (36.8)	32 (42.7)	0.64
Yes, less than daily	28 (8.6)	6 (8.0)	
No	178 (54.6)	37 (49.3)	
**Alcohol use disorder (AUDIT-C positive)***			
Yes	191 (58.6)	31 (41.3)	0.99
No	135 (41.4)	44 (58.7)	
**Past TB treatment**			
Yes	44 (13.5)	13 (17.3)	0.39
No	282 (86.5)	62 (82.7)	
**Self-reported health-seeking delay**			
<4 weeks	234 (73.8)	51 (69.9)	0.82
4-7.9 weeks	57 (18.0)	15 (20.6)	
8-11.9 weeks	15 (4.7)	5 (6.9)	
≥12 weeks	11 (3.5)	2 (2.70	
**Does anyone influence your health decisions?**			
Yes	219 (67.4)	50 (66.7)	0.91
No (only myself)	106 (32.6)	25 (33.3)	

*Alcohol use disorder defined as an AUDIT-C score ≥ 4 in men and ≥ 3 in women. All values represent the number and corresponding column percentage, unless otherwise specified. #P-value tests whether there is a difference across the two groups using Fisher's exact tests, Pearson's chi-squared tests, or Kruskal Wallis tests, as appropriate.

© 2022 Kerkhoff AD et al. *JAMA Network Open.*

eTable 3. Health Influences According to Latent Class Preference Group (N=326)

	**Overall**<br><br>**(n=326)**<br><br>**N (%)**	**“Time is Money” (n=192)**<br><br>**N (%)**	**“Privacy and convenience” (n=83)**<br><br>**N (%)**	**“Status quo” (n=51)**<br><br>**N (%)**	**P-value[#]**
**Does anyone influence your health decisions?**					
Yes	219 (67.4)	111 (57.8)	61 (74.4)	47 (92.2)	<0.001
No (only myself)	106 (32.6)	81 (42.2)	21 (25.6)	4 (7.8)	
**Which persons influence your health decisions?[#]**					
Family members (not spouse or partner)	205 (93.6)	105 (94.6)	56 (91.8)	44 (93.6)	0.77
Friends	158 (72.2)	84 (75.7)	47 (77.1)	27 (57.5)	0.039
Doctors/healthcare workers	138 (63.0)	88 (79.3)	39 (63.9)	11 (23.4)	<0.001
Spouse/partner	126 (57.5)	64 (57.7)	38 (62.3)	24 (51.1)	0.50
Religious leaders	118 (53.9)	66 (59.5)	35 (57.4)	17 (36.2)	0.022
Coworkers	105 (48.0)	53 (47.8)	33 (54.1)	19 (40.4)	0.37
Neighbors	99 (45.2)	50 (45.1)	31 (50.8)	18 (38.3)	0.43
**What are the best ways to reach you with health-related information?**					
Newspapers/magazines	195 (59.8)	100 (52.1)	52 (62.7)	43 (84.3)	<0.001
Radio	281 (86.2)	153 (79.7)	77 (97.8)	51 (100)	<0.001
TV	273 (83.7)	152 (79.2)	72 (86.8)	49 (96.1)	0.006
Billboards	259 (79.5)	142 (74.0)	69 (83.1)	48 (94.1)	0.004
Social media	229 (70.3)	126 (65.6)	60 (72.3)	43 (84.3)	0.031
Brochures	271 (83.1)	153 (79.7)	72 (86.8)	46 (90.2)	0.13
Health workers	308 (94.8)	181 (94.3)	79 (96.3)	48 (94.1)	0.78
Family	296 (91.1)	173 (90.1)	72 (87.8)	51 (100)	0.018
Friends/coworkers	275 (84.6)	162 (84.4)	64 (78.1)	49 (96.1)	0.012
Neighbors	254 (78.2)	148 (77.1)	59 (72.0)	47 (92.2)	0.013
Religious leaders	295 (90.8)	174 (90.6)	72 (87.8)	49 (96.1)	0.28
Teachers	309 (95.1)	178 (92.7)	81 (98.8)	50 (98.0)	0.07
Plays in the community	310 (95.7)	182 (94.8)	78 (95.1)	50 (100)	0.27

[#]n=219 participants who stated that at least one type of person other than themselves influenced health-related decisions.
[#]P-value tests whether there is a difference across the three groups using Fisher’s exact tests, Pearson’s chi-squared tests, or Kruskal Wallis tests, as appropriate.

© 2022 Kerkhoff AD et al. *JAMA Network Open.*

eTable 4. Simulated Shares of Preference (%) for a Health Facility Offering at Least One Enhanced Service Feature Under Three Different Implementation Scenarios According to Latent Class Preference Group*

	Overall (n=326)			"Time is Money" (n=192)			"Privacy and convenience" (n=83)			"Status quo" (n=51)		
	Scenario 1	Scenario 2	Scenario 3	Scenario 1	Scenario 2	Scenario 3	Scenario 1	Scenario 2	Scenario 3	Scenario 1	Scenario 2	Scenario 3
**Status quo**	47.5	45.3	34.3	47.8	44.6	35.4	46.2	44.4	28.6	48.5	49.6	39.2
**Single component strategy**												
**1. HCW is same sex**	44.5	42.3	33.4	46.9	43.8	36.3	38.9	37.2	24.8	44.4	45.4	36.5
**2. Extra weekday hours**	46.4	44.4	33.8	46.6	43.7	34.9	44.4	42.8	27.9	48.6	49.7	39.7
**3. Open Saturday**	54.1	52.0	40.9	54.5	51.4	42.1	54.8	52.9	37.1	51.5	52.6	42.2
**4. 2 hours shorter visit**	54.3	52.3	42.8	65.5	63.0	54.5	49.9	44.6	30.7	24.3	24.7	18.6
**5. 2 kilometers closer**	-	58.8	-	-	57.2	-	-	62.2	-	-	58.9	-
**6. Enhanced confidentiality**	61.0	59.2	34.3	61.2	58.8	35.4	68.1	66.2	28.6	48.5	49.4	39.2
**7. 30ZMW testing incentive**	62.0	59.9	49.5	64.7	61.7	53.4	58.9	56.8	41.5	56.8	57.9	48.1
**8. 60ZMW testing incentive**	70.7	68.9	59.4	74.0	71.5	64.1	67.9	66.0	51.3	63.0	64.0	54.9
**9. Test results by phone**	34.1	32.8	29.3	55.8	53.8	48.6	5.0	4.4	2.7	0.0	0.0	0.0
**10 Same day test results (+3 hours total visit time)**	69.9	66.3	63.8	69.2	67.4	63.6	83.8	79.2	75.4	50.1	41.5	45.8
**11. Same day test results (+1 hour total visit time)**	74.1	72.0	68.6	78.4	77.2	73.8	87.0	84.5	79.0	36.7	32.2	32.0
**Multi-component strategy**												
**12. Confidentiality + 2hr shorter total visit time**	64.0	63.3	54.8	73.3	72.4	65.3	66.8	65.7	52.6	24.3	25.1	18.9
**13. Confidentiality + 2 kilometers closer**	-	69.1	-	-	67.9	-	-	78.6	-	-	58.0	-
**14. Enhanced confidentiality + Open Saturdays**	66.4	64.9	55.3	66.6	64.5	56.5	75.1	73.5	60.1	51.5	52.3	42.7
**15. Enhanced confidentiality + 60ZMW testing incentive**	77.3	76.6	69.0	79.1	78.1	72.3	82.4	81.6	70.2	62.4	63.0	54.6
**16. Same day test results (+3 hours) + Open Saturday**	72.2	69.1	66.5	71.5	70.2	66.1	86.2	82.5	78.8	52.1	43.4	47.6
**17. Same day test results (+3 hours) + 2 kilometers closer**	-	72.2	-	-	72.5	-	-	87.0	-	-	46.6	-
**18. Same day test results (+3 hours) + Enhanced confidentiality**	75.0	72.3	70.3	74.6	73.3	70.5	91.4	89.2	85.3	49.4	41.0	45.4
**19. Same day test results +2 hours shorter total visit time (1 hour total)**	76.1	78.6	71.6	87.7	88.8	84.6	86.9	93.5	78.3	15.1	15.7	11.9
**20. Same day test results (+3 hours) + 60ZMW testing inventive**	79.3	83.5	74.7	80.4	90.1	76.5	90.3	96.9	83.7	57.1	37.0	53.6

***Three different implementation scenarios were simulated using the simulated shares of preference (logit rule) method:**

© 2022 Kerkhoff AD et al. *JAMA Network Open.*

In scenario 1 (S1), the input parameters were based on the features of a typical TB diagnostic facility at a first level health facility in Lusaka, Zambia. The “usual care” health facility was assumed to be 2 kilometers from a participant’s home, require three hours spent at the clinic waiting and undergoing evaluation (based on the median amount of time cited by survey participants on their date of TB diagnosis), only be open during typical business hours Monday through Friday, be a facility where an individual may be known or recognized, not offer sex-concordant health care providers, not offer financial incentives for undergoing TB testing, and require patients to return on a different day to collect their TB test results. The “enhanced facility” was assumed to have the same features as the “usual care” facility with the exception that it offered one or more improved service features.

In scenario 2 (S2), we explored the effect of an implementation context in which the TB diagnostic facility was less physically accessible (e.g., further away from a person’s home and longer total times spent waiting and being evaluated). The “usual care” health facility was assumed to be 4 kilometers from a participant’s home (twice as far as S1), require five hours spent at the clinic waiting and undergoing evaluation (2 hours longer than S1), only be open during typical business hours Monday through Friday, be a facility where an individual may be known or recognized, not offer sex-concordant health care providers, not offer financial incentives for undergoing TB testing, and require patients to return on a different day to collect their TB test results. The “enhanced facility” was assumed to have the same features as the “usual care” facility with the exception that it offered one or more improved service features.

In scenario 3 (S3), we explored the effect of an implementation context in which enhanced facility features could only be feasibly implemented in select, centralized facilities (e.g., further away from a person’s home). The “usual care” health facility was assumed to be 2 kilometers from a participant’s home, require three hours spent at the clinic waiting and undergoing evaluation (based on the median amount of time cited by survey participants on their date of TB diagnosis), only be open during typical business hours Monday through Friday, be a facility where an individual may be known or recognized, not offer sex-concordant health care providers, not offer financial incentives for undergoing TB testing, and require patients to return on a different day to collect their TB test results. The “enhanced facility” was assumed to have the same features as the “usual care” facility with the exception that it was assumed to be 4 kilometers from a participant’s home (e.g., twice as far away as the status quo clinic) and offered one or more improved service features that were evaluated during the DCE.

© 2022 Kerkhoff AD et al. *JAMA Network Open.*

eTable 5. Simulated Shares of Preference Using Two Different Models for a Health Facility Offering at Least One Enhanced Service Feature Using Two Different Estimation Methods According to Latent Class Preference Group

	**Overall (n=326)**		**"Time is Money" (n=192)**		**"Status quo" (n=51)**		**"Privacy and convenience" (n=83)**	
	**SOP Method**	**RFC Method**	**SOP Method**	**RFC Method**	**SOP Method**	**RFC Method**	**SOP Method**	**RFC Method**
**Status quo**	47.5	42.5	47.8	42.8	48.5	43.7	46.2	41.1
**1. HCW is same sex**	44.5	42.5	46.9	44.1	44.4	43.2	38.9	38.5
**2. Extra weekday hours**	46.4	43.9	46.6	44.0	48.6	45.8	44.4	42.3
**3. Open Saturday**	54.1	48.8	54.5	49.1	51.5	47.6	54.8	49.1
**4. Enhanced confidentiality**	61.0	54.7	61.2	55.2	48.5	45.6	68.1	59.1
**5. 30ZMW testing incentive**	62.0	56.7	64.7	59.3	56.8	52.2	58.9	53.5
**7. 60ZMW testing incentive**	70.7	65.3	74.0	68.6	63.0	58.3	67.9	61.8
**8. Test results by phone**	34.1	32.4	55.8	51.8	0	0.1	5.0	7.6
**9. Same day test results (+3 hours total visit time)**	69.9	68.2	69.2	67.9	50.1	48.8	83.8	81.1
**10. Same day test results (+1 hour total visit time)**	74.1	72.8	78.4	77.1	36.7	36.3	87.0	85.2
**11. Enhanced confidentiality + Open Saturdays**	66.4	62.3	66.6	62.7	51.5	49.7	75.1	69.1
**12. Enhanced confidentiality + 60ZMW testing incentive**	77.3	75.7	79.1	77.8	62.4	60.6	82.4	80.0
**13. Same day test results (+3 hours) + Open Saturday**	72.2	71.2	71.5	70.7	52.1	51.1	86.2	84.8
**14. Same day test results (+3 hours) + Enhanced confidentiality**	75.0	74.2	74.6	74.0	49.4	48.8	91.4	90.2
**15. Same day test results (+3 hours) + 60ZMW testing inventive**	79.3	79.3	80.4	80.4	57.1	57.1	90.3	90.5

Abbreviations: HCW - health care worker; RFC - randomized first choice; SOP – shares of preference (logit rule); ZMW Zambian Kwacha

Input parameters for both models were the same and were based on the features of a typical TB diagnostic facility at a first level health facility in Lusaka, Zambia. The "usual care" health facility was assumed to be 2 kilometers from a participant's home, require three hours spent at the clinic waiting and undergoing evaluation (based on the median amount of time cited by survey participants on their date of TB diagnosis), only be open during typical business hours Monday through Friday, be a facility where an individual may be known or recognized, not offer sex-concordant health care providers, not offer financial incentives for undergoing TB testing, and require patients to return on a different day to collect their TB test results. The "enhanced facility" was assumed to have the same features as the "usual care" facility with the exception that it offered one or more improved service features.

© 2022 Kerkhoff AD et al. *JAMA Network Open.*

eReferences

1. Hauber AB, González JM, Groothuis-Oudshoorn CGM, et al. Statistical Methods for the Analysis of Discrete Choice Experiments: A Report of the ISPOR Conjoint Analysis Good Research Practices Task Force. *Value Health*. 2016;19(4):300-315. doi:10.1016/j.jval.2016.04.004

2. Sawtooth Software. Lighthouse Studio Manual. Sawtooth Software, Provo; 2022.

3. Weller BE, Bowen NK, Faubert SJ. Latent Class Analysis: A Guide to Best Practice. *J Black Psychol*. 2020;46(4):287-311. doi:10.1177/0095798420930932

4. Zhou M, Thayer WM, Bridges JFP. Using Latent Class Analysis to Model Preference Heterogeneity in Health: A Systematic Review. *Pharmacoeconomics*. 2018;36(2):175-187. doi:10.1007/s40273-017-0575-4

5. Orme BK. *Getting Started with Conjoint Analysis: Strategies for Product Design and Pricing Research. Madison, Wis: Research Publishers LLC,*.2010.

6. Sawtooth Software. The CBC/HB System - Technical Paper v5.6. Sawtooth Software, Provo; 2021.

7. Cheng J, Pullenayegum E, Marshall DA, Marshall JK, Thabane L. An empirical comparison of methods for analyzing correlated data from a discrete choice survey to elicit patient preference for colorectal cancer screening. *Bmc Med Res Methodol*. 2012;12(1):15-15. doi:10.1186/1471-2288-12-15

© 2022 Kerkhoff AD et al. *JAMA Network Open.*
